# Investigation of the usage of machine learning to explore the impacts of climate change on occupational health: a systematic review and research agenda

**DOI:** 10.3389/fpubh.2025.1578558

**Published:** 2025-06-16

**Authors:** Guilherme Neto Ferrari, Gislaine Camila Lapasini Leal, Paulo Cesar Ossani, Edwin Vladimir Cardoza Galdamez

**Affiliations:** ^1^Production Engineering Department, State University of Maringá, Paraná, Brazil; ^2^Department of Informatics, Postgraduate Program in Computer Science, State University of Maringá, Paraná, Brazil

**Keywords:** occupational health and safety, heat stress, climate change, machine learning, supervised learning

## Abstract

Occupational accidents can be potentialized by factors related to the workplace or the environment, such as climatic conditions. Air temperature, wind speed, and humidity can be used to monitor occupational heat stress, leading to cramps, exhaustion, stroke, and even death. Under the climate change scenario, measuring these variables is fundamental to developing adaptation strategies for maintaining the workers’ well-being. However, when dealing with this high data volume from distinctive factors, traditional techniques are insufficient to extract all information effectively. Therefore, computational intelligence and data analytics tools can enhance data processing and analysis. Machine learning techniques have been successfully applied to occupational health and climate contexts. This paper explores the literature regarding applying these techniques to investigate the effects of climate change on occupational health. We conducted a systematic review through five scientific databases guided by three research questions, resulting in 24 selected papers. 75% of the papers screened used primary data collected from wearable sensors to monitor the well-being of workers, where we identified a trend of using supervised machine learning techniques, especially classification and regression algorithms, such as SVM, RF, and KNN. The remaining focus is on using secondary data from national databases to investigate the risk, with a trend of using feature selection techniques and classification tasks. Considering this topic is relatively new, we developed an agenda to guide future research, with suggestions to follow the trends found in this review and highlight the potential of expanding to multiple future research paths.

## 1 Introduction

The lack of safety and health measures in the workplace can lead to increased occupational accidents and illnesses and a consequential drop in productivity and work capacity (1, 2). Often, studies that focus on preventing and reducing accidents aim at understanding their root causes, which can be related to the type of activity that is being done, e.g., work at heights (3), or due to the lack of safety policies such as protective equipment (4).

External factors, precisely climatic conditions, can directly influence the occurrence of occupational accidents and illnesses (5). Air temperature, humidity, and wind speed are often considered when calculating heat stress, a heat-induced strain caused by exposure to high temperatures without the precautions necessary (6–8). Heat stress can lead to several consequences, ranging from dizziness, fatigue, and exhaustion to heat-related illnesses, heat strokes, and death (9–12).

Heat stress adds a new layer to studies on occupational health and safety (OHS). When considering the current weather scenario worldwide, climate change endangers the survival and integrity of life, and weather conditions should not be ignored when studying OHS (13, 14). Any OHS risk potentialized by weather conditions can be directly connected to climate change and will be impacted by it (8).

Heat stress is one of the most frequently studied climate change effects to occupational health and safety, as it is easily measured by a range of different methodological approaches (6). However, climate change is not limited to higher temperatures, it also affects the transport and dispersion of particles in the air, making the workers more exposed to air pollution; heat stress also modifies the body absorption of chemicals, making the workers more vulnerable to gases and pesticides; other environmental characteristics are changing and becoming more dangerous, such as the UV radiation and extreme weather events that are becoming more frequent; the workers can also be more exposed to vector-borne and zoonotic diseases as a consequence to changes in rain and drought and to extreme weather events (15–19).

Therefore, to tackle the climate change impacts on worker’s health, new variables must be considered in addition to OHS. The more data included, the more precise is the investigation regarding indirect impacts, however, the downside is the generation and processing of a large volume of data. Often, traditional approaches with statistical models face some limitations regarding exploring the relationships between the accidents and the causes; the complexity of these relationships may be challenging to model appropriately, making it difficult to gather the whole scenario of possibilities. This way, a more sophisticated approach is required, using computational intelligence tools (20, 21).

Computational intelligence, more specifically data analytics tools, such as machine learning (ML), have already been used in studies on OHS, providing a more complete view of the problem and its root causes (22). ML techniques make it possible to accurately predict whether an injury might happen to an employee (23). The selection of essential features for modeling is fundamental; by knowing the nature of the accident and type of exposure, we know the most frequent type of accidents and their results (24). However, these papers do not consider the specifications of including weather conditions into the equation. On the other hand, computational intelligence can also be seen in the context of climate change. Rolnick (25) presented a comprehensive review of the possibilities of using machine learning to approach climate change issues from mitigation and adaptation perspectives and as a tool for action. However, this case lacks the investigation of the effects of climate change specifically on the working population. There is available literature that deals with these three topics in pairs, however, is there evidence of the usage of machine learning to assess and monitor the effects of climate change on occupational health?

Therefore, the main objective of this paper is to explore the literature regarding the use of ML techniques to investigate the effects of climate change conditions on occupational safety and health. The purpose of the paper is to understand the role ML has on the monitoring of occupational health when dealing with the effects of climate change. We hope to understand the type of data used in these approaches, the type of ML technique used in each case, and its contributions to the topic. To achieve this goal, we developed a systematic literature review on both journal publications and conference papers, aiming to identify information relevant to understanding the scientific scenario of how this issue is being addressed. The selected papers were processed, and we collected data regarding the method used, the type of data used, the type of ML technique being applied, and the results. With these results, we develop a research agenda to organize and present directions and priorities to guide research efforts; it enables researchers to make meaningful contributions (26). Our agenda focuses on two different categories of papers found in our review, discussing research trends and gaps, some limitations, and our suggestions.

## 2 Methods

We developed a systematic literature review to explore the literature regarding this research topic. We followed the Preferred Reporting Items for Systematic Reviews and Meta-Analyses (PRISMA) method, which is composed of three main steps: identification, screening, and inclusion.

The first step, identification, involves the definition of the Search Strategy, which includes the selection of the search terms, the definition of a time frame of our search and the specification of languages included. We followed the PICOS framework to define our search terms. Our Population regards the workers, without any restriction of the type of work, workplace, or location; our Intervention regards the usage of machine learning, including supervised or unsupervised learning, not limited to any kind of technique or approach; our Comparison regards the application of these machine learning techniques considering the context of occupational health, excluding any studies that tackles the issue as a generic and broad public health problem; the Outcome we are searching regards the effects of climate change, which we included terms related to temperature and heat stress as they are commonly studied in the occupational health context.

Finally, our Study Design is a systematic review in five well-known and frequently used databases: ACM Digital Library, IEEE Xplore, PubMed, Scopus, and Web of Science. Considering the PICOS framework, our paper is based on four main concepts: climate change, health and safety, work/labor/occupation, and machine learning. Therefore, the keywords related to each term are presented in Table 1. For our search strategy, we did not include any limitations regarding the time frame, and we considered both journal and conference papers. We are not interested in reviews, meta-analyses, mapping, or any research that gathers and discusses the topic based on the literature and not based on collected data. The only filter applied to each database was related to the language, where only publications in English were considered. In addition to these databases, we also conducted a snowball search in the final included articles, both forward and backward snowballing. The first step was validated through a research protocol sent to other researchers. The protocol followed the PRISMA protocol guidelines.

**Table 1:** Relevant terms and keywords.

Terms	Keywords
Climate change	“climate change”; “global warming”; “heat stress”; “temperature”;
Health and safety	“health”; “safety”; “well-being”; “accident”; “injury”; “illness”; “incident”
Occupational	“occupational”; “labour”; “worker*”
Machine learning	“machine learning”; “supervised learning”; “unsupervised learning”

The second step is the screening. The screening process involves the definition of Eligibility Criteria to guide our selection of papers that fit our scope. For this selection and evaluation of eligibility, we defined six exclusion criteria: EC1: Not published in conferences or journals; EC2: Not applied in the context of occupational health and safety; EC3: Does not include the climate context; EC4: Duplicated papers; EC5: Papers that are not available online and freely; EC6: Papers that do not use primary or secondary data to approach the issue.

The eligibility criteria were considered in two steps: the first when reading titles, abstracts, and keywords, and the second when reading the introduction and conclusions. The selected papers were approved for the next step.

The last step of the review process was the inclusion. This step involves the full reading of the selected papers. To ensure that the selected papers would contribute to our review, we defined eleven Quality Assessment criteria based on Dyba et al. (27). These criteria consider four categories: the Quality of the Reporting, regarding clarity of the study’s aims, objectives, and context; the second is Rigor, which assesses the adequacy of research design, sampling, data collection, and analysis methods. The third is Credibility, which examines the researcher-participant relationship and whether the study provides clear, credible, and justified findings and conclusions. The last is Relevance, which considers whether the study adds value to research or practice. These criteria were graded by answering “yes” or “no” questions. This assessment was used to measure how confident the reviewers were that the assessed studies’ findings could contribute to our review. The assessed and approved papers went through the Extraction Process, where we gathered the relevant information of the papers for our analysis, including title and authors of the papers, year of publication, methodology of the paper, OHS data and its source, type of data (primary or secondary), machine learning method used, results accomplished, climate change effect studied, country where the research was conducted, and workplace studied. As our findings are limited to 24 papers, our Data Analysis and Synthesis Method are limited to reading and extracting relevant information, grouping the read papers into categories to facilitate the comparison and analysis.

## 3 Results

**Figure 1:**
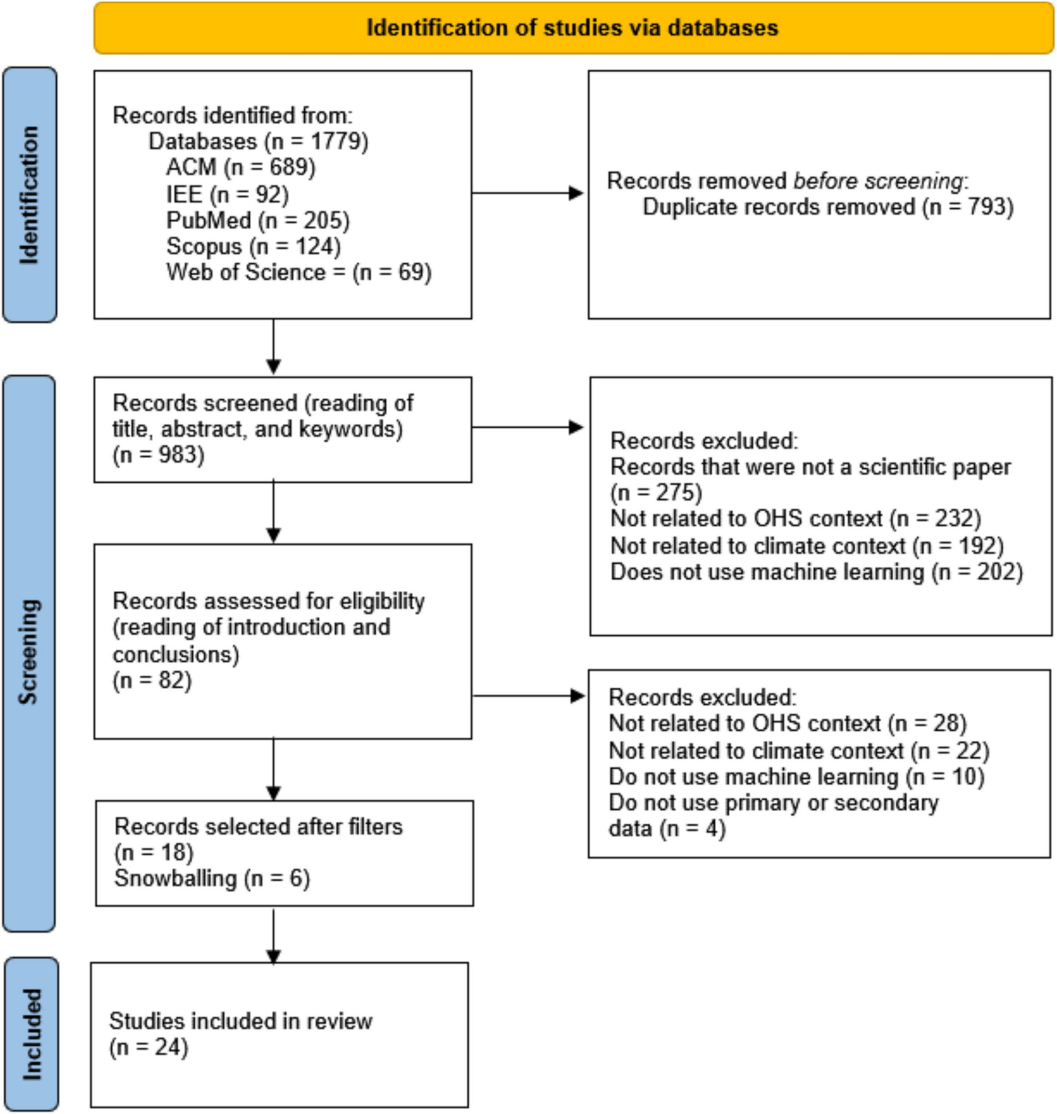
Selection process of the papers.

The search process was done in December 2024 and resulted in 1779 publications. After reading the titles, keywords, and abstracts, we excluded duplicates and applied the exclusion criteria, reducing the number to 82 papers. After reading the introductions and conclusions, we reduced it even further to 18 publications. From the 18 final articles, we applied the snowballing process, both backward snowballing and forward snowballing, the former consulting the references used in the previous papers, where we found three more relevant publications, and the latter checked for new publications that cited these selected papers, where we included three more publication. Therefore, in the end, we had 24 publications to be fully read. During the reading, the quality of the papers was assessed to ensure their contributions to our study. This whole process is presented in Figure 1.

When reading the papers, we noticed that the papers included in our screening presented two different approaches to collect and explore occupational data. The first approach uses primary data collected through some sensor or wearable device that gathers and monitors essential variables, such as from the worker’s body or the environment. The second approach uses data from secondary sources, such as national records of occupational injuries and illnesses. To better explore the 24 selected papers, we grouped them into two categories related to these two approaches. With this, we have 18 papers in the first category and 6 in the second, which will be more detailed in the remaining of our paper.

The first point to discuss regarding the results is that the majority of the papers discuss the effects of heat stress, even with other climate change keywords included in the search process. This trend was observed in previous reviews (6). The 24 papers are presented in Table 2 by their category, title, year of publication, and source. We added an ID following the categories, where P refers to primary data study, and S refers to secondary data study, which is used for later reference in our paper.

**Table 2:** Articles included in the systematic review, their respective year, and source of publication.

ID	Title	Year	Source
Primary data			
P01	Leveraging knowledge from physiological data: On-body heat stress risk prediction with sensor networks	2013	IEEE Transactions on Biomedical Circuits and Systems
P02	Towards decisive garments for heat stress risk detection	2016	2016 ACM International Joint Conference on Pervasive and Ubiquitous Computing
P03	Intelligent Wearable Occupational Health Safety Assurance System of Power Operation	2018	Journal of Medical Systems
P04	Application of Wearable Biosensors to Construction Sites. II: Assessing Workers’ Physical Demand	2019	Journal of Construction Engineering and Management
P05	Accuracy of Algorithm to Non-Invasively predict core body temperature using the Kenzen Wearable Device	2021	International Journal of Environmental Research and Public Health
P06	Assessing occupational risk of heat stress at construction: A worker-centric wearable sensor-based approach	2021	Safety Science
P07	Heat Stroke Prevention in Hot Specific Occupational Environment Enhanced by Supervised Machine Learning with Personalized Vital Signs	2022	Sensors
P08	Developing Prediction Models for Monitoring Workers’ Fatigue in Hot Conditions	2023	Computing in Civil Engineering
P09	Long-Short-Term-Memory-Based Deep Stacked Sequence-to-Sequence Autoencoder for Health Prediction of Industrial Workers in Closed Environments Based on Wearable Devices	2023	Sensors
P10	Machine Learning Approach to Model Physical Fatigue during Incremental Exercise among Firefighters	2023	Sensors
P11	A Classification Model Using Personal Biometric Characteristics to Identify Individuals Vulnerable to an Extremely Hot Environment	2024	Journal of Management in Engineering
P12	A machine learning-based forecasting model for personal maximum allowable exposure time under extremely hot environments	2024	Sustainable Cities and Society
P13	A System for Individual Environmental Risk Assessment and Management with IoT Based on the Worker’s Health History	2024	Applied Sciences
P14	Enhancing Workplace Safety through Personalized Environmental Risk Assessment: An AI-Driven Approach in Industry 5.0	2024	Computers
P15	Forecasting personal heat strain under extremely hot environments: Utilizing feature importance in machine learning	2024	Engineering Applications of Artificial Intelligence
P16	Identification of Fatigue using Data Analytics and Machine Learning to Improve Worker’s Health	2024	International Conference on Cognitive Robotics and Intelligent Systems
P17	Individual Environmental Risk Assessment and Management in Industry 4.0: An IoT-Based Model	2024	Applied System Innovation
P18	Worker-centric heat strain analysis: Integrating physiological signals with ensemble learning and domain adaptation	2024	Automation in Construction
Secondary data			
S01	Exploring Fatalities and Injuries in Construction by Considering Thermal Comfort Using Uncertainty and Relative Importance Analysis	2021	Int. J. Environ. Res. Public Health
S02	External Climate Data Extraction Using the Forward Feature Selection Method in the Context of Occupational Safety	2022	Lecture Notes in Computer Science
S03	Integrated feature selection and classification algorithm in the prediction of work-related accidents in the retail sector: A comparative study	2022	International Conference on Optimization, Learning Algorithms and Applications.
S04	Predicting Maximum Work Duration for Construction Workers	2022	Sustainability
S05	Severity Analysis for Occupational Heat-related Injury Using the Multinomial Logit Model	2024	Safety and Health at Work
S06	Unpacking Occupational Health Data in the Service Sector: From Bayesian Networking and Spatial Clustering to Policy-Making	2024	Mathematical Geosciences

As we can see in Table 2, the publications regarding this topic are very recent, 6 out of 21 were published in the last 5 years, and the older one was published 11 years ago. The year 2024 was the most frequent one among the included papers, so we can notice that there is a trend of growth in popularity on this topic over the last years.

The source of the papers was very heterogeneous with 21 unique sources. We can notice that most of the publications are from journals, while three are from conferences, all three being computing-related conferences. It is common for computer science and related areas scholars to publish more in conferences than journals, which can be due to a faster acceptance process and a legitimate path to formally evaluate research (28, 29). The remainder of this section is dedicated to each of these two categories, we are going to explore the data extracted for each article, defining their methods, the type of data used, and the machine learning approach, and discuss our findings.

### 3.1 Primary data

Most papers included in this review, 18 out of the 24, fit this category, and they share a similar method to collect data: using sensors on wearable devices, which means that the information used to estimate heat stress is collected from the specific workplace studied, using real data from workers, such as the metabolic and physiological response of the workers’ body to the climate exposure. However, they differ in the kind and volume of data they are gathering and the methodological approach to process and analyze them, that is, the ML technique applied.

Regarding the sensors, all papers used a wearable device to collect workers’ physiological data and environmental conditions. The specifics of the wearable sensors change for each paper. P01 focuses on a non-invasive body sensor system composed of a network of nodes placed in the helmet, the jacket, and the trousers that gathers and monitors real-time data such as temperature, CO2 levels, heart pulse, and acceleration (30). Following this path of non-invasive devices, in P02, the authors discuss the development of a sensor shirt that collects firefighters’ body temperature data (31).

The search for a non-invasive and non-intrusive sensor to monitor the health state of workers is a trend. Some of the papers focused on sensors worn on the wrist, such as smartwatches and bracelets. The study presented by P03 discussed the development of a watch sensor that monitors the environmental conditions and physiological state of power operators. Different variables, including vital signs such as electrocardiogram, pulse, body temperature, blood oxygen, and blood pressure, are measured by the watch and sent to the server, where the computing and calculations of fatigue level are going to happen, through a portable information processing gateway, similar to a walkie-talkie, that is clipped to the arm of the worker (32).

There is a worry about not overwhelming the worker with heavy sensors and not hindering their activities, focusing on smaller, lighter devices. Paper P04 used a wristband device collected the photoplethysmogram (PPG), electrodermal activity (EDA), and skin temperature (ST), which were used to estimate the physical demand of the workers (33). In P05, another version of the wearable devices is attachable to the arms of the workers, it collects the heart rate of the user, as well as the skin temperature and humidity; the sensor has a mobile application to gather qualitative data about the worker using the device, such as age, height, and body mass, among others (34). P06 is similar to P04, it used a wristband device to measure a worker’s physiological data as PPG, EDA, and ST, which were used to estimate the heat stress in P06 (35).

In P07, the authors developed a sensor that is fixed in the upper arm of the worker with a belt; the device is composed of two PPG sensors, one that has contact with the skin surface and one that is non-contact, to take into account the movements that the user might do during labor that may create some noises in the heart rate measurements (36). P08 used an Electromyography (EMG) device to monitor the muscle activation of the upper arm during different activities, along with a biosensor attached to the chest to monitor heart rate (HR) and heart rate variability (HRV) (37). P09 used a bracelet that gathered information about the oxygen saturation level in the worker’s blood, the pressure exerted by the worker, and body temperature measurements (38).

The fatigue identification is also the motivation for P10, the paper monitors the physical exertion through a heat rate monitoring belt and a telemetric portable gas analyzer that measures the respiratory gas exchange of a controlled activity that simulates construction activities (39). The same team of authors developed P11 and P12, which have similar data collection and investigation approaches. The papers used the popular smartwatch Apple Watch to collect heart rate and a wireless in-ear monitoring system that gathers the eardrum temperature and is used as a reference for core temperature. The measurement of personal variables such as height, weight, body mass index, and body fat mass, among others, through body composition analyses (40, 41).

A similar thing can be observed in P13 and P14, two papers by the same team of authors regarding the same system of sensors that is clipped to the employee’s clothes to collect environmental data, including dust, noise, ultraviolet radiation, illuminance, temperature, humidity and the presence of flammable gasses in the work settings. In P13 the authors delve into the development of the system and its technical functionalities (42). Finally, in P14, the authors used the systems by generating data to test the models (43).

P15 is from the same authors that wrote P11 and P12, it uses the same methodological approach as the first two papers (44). P16 uses data from wearable sensors and cognitive evaluations to diagnose fatigue in construction workers (45). P17 was written by the same team of authors that wrote P13 and P14. In P17 they present the initial idea of the model and how it would work (46). Finally, in P18, besides the wristband-type sensor that gathered EDA, PPG, and ST data, the author also used a chest sensor to monitor the respiration rate during the experiment that simulated and estimated the heat strain of construction workers’ activities (47).

The papers also differentiate between the type of work setting and the worker’s occupation being studied. Construction has the most applications, with eight papers (P04, P06, P08, P11, P12, P15, P16, and P18). P04 focused on different tasks, labeling them as light, moderate, or heavy workload (30); P06 focused on roofing and materials-handling tasks (35); P08 simulated manual handling present in construction tasks by performing the dumbbell curling task (37); P11, P12, and P15 used a treadmill to simulate an activity with a metabolic rate similar to the workload of construction workers (40, 41, 44); P16 did not specify the construction task (45) and P18 used a virtual reality headset to simulate the logging task, an activity common on construction settings in the United States of America (47).

The remaining papers focused on different scenarios. P01 focused on military workers responsible for the disarming and disposal of explosive threats in military settings (30); P02 and P10 focused on firefighters (31, 39); P03 talked about power operators (32); P07 studied train maintenance factory (36); and P09 concentrated on the health of workers in confined workspaces (38). Finally, papers P13, P14, and P17 did not specify the work settings, as they were developing and proposing a new model and sensor system and used created data to test its functionalities (42, 43, 46). P05 also used a treadmill to estimate body temperature. However, the authors did not specify if the activity replaced a task from a specific work setting (34).

Although all papers focus on occupational health and safety effects, not all of them actively collected data from real-life work settings. Some papers did not use the sensor on real subjects, such as P03 (32), which only discussed the development of the sensor, and P13 (42), P14 (43), and P17 (46), which presented a new sensor and used simulated data to test the system. Other papers used subjects with no field experience in their experiments, such as P06 (35), which used undergraduate and graduate students to simulate construction tasks in a controlled environment. P11 (40), P12 (41), and P15 (44) used volunteer students to perform experiments inside an environmental chamber where climatic variables are controlled. Finally, P05 (34) and P08 (37) did not specify the selection of the volunteers.

On the other hand, P01 gathered data from military trials, where subjects underwent three mission-like protocols, each with a different in-suit cooling (30); P02 used data collected from a fire-fighting training session with activities under normal and extreme conditions (31); P04 collected data from a real construction site, gathering data from the wearable sensors and taking footage of all the experiments performed (33); P07 used the wearable sensors to collect real data from workers for all work day for over a month (36); P09 monitored over forty workers operating in closed space (38); P10 applied the study on twenty-four individuals from a fire brigade (39); P16 uses a dataset from construction workers (45); and P18 used participants with field experience in construction to conduct the experiment (47).

Regarding the machine learning application, most papers used more than one machine learning method, most of which compared results and metrics between algorithms. A range of different algorithms includes classification, regression, and clustering techniques. A few algorithms were more frequent than others. Support Vector Machine (SVM) was used by 10 papers with different types of SVM. In P03, the authors selected the Directed Acyclic Graph-Support Vector Machine (DAG-SVM) (32). In P04, the authors used a Gaussian kernel SVM (33). In P07, a linear SVM was used (36). In P08 and P16, a simple SVM was used (37, 45). In P09, the authors used different types of SVM: a method with one-class SVM with linear kernel, a second method with one-class SVM with RBF kernel, a third method with one-class SVM with poly kernel, and finally, a method with handcrafted features and one-class SVM (38). P10 used different kernel functions for the SVM: linear, quadratic, cubic, and Gaussian (39). P11 and P14 used a variation of the SVM method: Support Vector Classification (SVC) (40, 43). Finally, P18 included a simple SVM, an SVM with a Gaussian kernel, and an SVM with a Polynomial kernel (47). The second most used method was Random Forest (RF), present in 8 papers (34, 35, 37, 39–41, 44, 45) followed by K-Nearest Neighbor (KNN), in 7 (33, 36, 37, 39, 43, 45, 47), eXtreme Gradient Boost (XGBoost) in 6 (34, 40, 41, 43–45), and Multi Layer Perceptron (MLP) in 5 (33, 40, 41, 44, 47).

### 3.2 Secondary data

The remaining six papers in this study fit this category; they all used some secondary data to develop their research. In the first category, the papers tackle the issue regarding a specific workplace, collecting personal data from a few workers. In the second category, they analyze the climate issue affecting a whole country. Some focus on specific industry sectors, while others discuss the effects on occupational health for all the workers from that country. This approach has some limitations, as the data used does not include the physiological and metabolic response of the workers’ body to climate variation, it depends exclusively on dataset regarding the occupational accidents or injuries along with a dataset regarding the climate. However, this approach is valid, as it is one of the most used in the literature when dealing with the effects of climate change on occupational health (6), therefore, its application in the context of machine learning must also be studied.

There are some similarities regarding the type of data used; most used are national occupational health databases and weather information. S01 used a Korean construction database of occupational fatal incidents and injuries and climate data that matches the date and location of the accidents with information regarding air temperature, relative humidity, and wind speed (48). In S02, the authors only collected meteorological data and used it to improve the accuracy of predictive models for occupational accidents. However, no data was gathered regarding the recording of injuries or accidents (49). The authors of S03 used data from five processed databases: (i) accident records; (ii) Ergonomic Workplace Analysis (EWA) of a specific retail company; (iii) Hazard Identification and Risk Assessment (HIRA), a detailed assessment of risk for each task performed by the company; (iv) climatic variables that match the data and place of the accidents records; (v) holiday records (50). In S04, the authors did not use historical records of accidents or injuries. However, they created simulated data about construction workers and calculated the average temperature according to meteorological data from the Hong Kong Observatory (51). In S05, the authors analyzed historical heat-related injury reports from the Occupational Safety and Health Administration (OSHA) and combined them with weather information (52). Finally, S06 used over seventy thousand occupational health surveillance tests from a Spanish database (53).

Regarding the industry sector that was the focus of the study, there were a few trends: two papers used construction worker data, S01 used real records (48), and S04 simulated construction worker data (51). Similarly, in two papers focused on the retail sector, S02 did not use any real record of retail workers but considered the specifics of the sector to conduct their work, while S03 used accident records. S05 is the only paper in the review that uses every record of occupational injury in a country, specifically heat-related injuries. Moreover, S06 focuses on the service sector. On the other hand, the climatic variables databases used by the papers were all similar. Every paper used a national database to calculate the meteorological aspect, while in the first category of papers, most papers collected their environmental variables using different devices.

Finally, regarding the machine learning approach, we identified a trend of using feature selection methods in some papers. In S01, the authors applied a Neural Network to calculate the variables of relative importance to identify the most influential environmental factors based on thermal comfort that affect injury and accidents; the results showed that air temperature and relative humidity are the most important factors that influence injury and fatal accidents. In S02, the authors applied the wrapper method Forward Feature Selection (FFS) to select the most relevant feature out of twenty. The relevance was measured according to the capability of predicting the air temperature through a Linear Regression Model. Four were considered significant: water temperature, dawn, night, and summer. In S03, the team of authors is the same as in S02, and they used a similar feature selection approach. They compared the FFS and Chi-Square methods to verify the relevant features concerning their capability of predicting the occurrence of accidents through 3 different prediction algorithms: RF, SVM, and Naive Bayes. In S05, the authors also included a process of variable extraction and selection. They used text mining to heat-related injuries (HRI) records to find the most frequent keywords in the accident descriptions, and using different machine learning algorithms such as Classification and Regression Trees, Bagging, Random Forest, and Bayesian Additive Regression Trees, they selected the essential variables to explore the relation between impact factors and HRI severity.

The remaining 2 papers did not use feature selection methods. In S04, the authors developed a new Linear Regression model to predict the maximum working duration of construction workers according to the average temperature of the period. In S06, the authors used an unsupervised Bayesian network to uncover the relationship between 48 variables, and then they clustered these variables into subgroups for analysis using an agglomerative hierarchical clustering algorithm. Finally, they constructed a supervised Bayesian network, which allowed the identification of the clusters with a higher impact on occupational health.

## 4 Discussion

Discussing some trends and their lack in these 24 papers is possible. The first trend is that most papers used primary data gathered from wearable devices. This category of study includes the two oldest papers included in this review, one from 2013 and the other from 2016, which shows that the worry of creating a way to monitor real-time occupational data has existed for more than a decade and, as the technology advances, this type of study gets more frequent, with one-third of the selected papers being from 2024.

During this period, not only the technology of the wearable device has advanced - we can notice that the wearable device described in P01 is a whole-body suit (30), while in papers like P14, the device is small and capable of gathering different environmental data, such as heat, dust, noise, and radiation (43) - but also the data handling and processing, making this approach more accessible, where the first two papers firstly discussed heat-related occupations, being anti-bomb military personnel and firefighters (30, 31). In contrast, the newest papers include other workplaces, such as construction (33, 37, 40, 41, 44, 45, 47), which are activities sensible to heat and other climatic conditions as well (6).

We noticed a trend among the primary data studies regarding the machine learning method. This type of paper collected physiological data with wearable devices and compared the values of temperature or heat stress with another method of measuring, such as core temperature measured with an ingestible telemetric temperature pill or tympanic temperature or by comparing with tabled heat stress indexes calculated with climatic variables controlled in the experiments. Different machine learning methods, mainly classification algorithms, were used in this comparison, where the higher accuracy indicated by these methods, the better the device was in correctly identifying the worker’s heat strain. There is a trend in using SVM, RF, and KNN.

Another trend noticed was that heat was the main explored impact on occupational health. Almost all papers collected some temperature measures, be it from the environment, such as air temperature, or the worker’s body, such as skin temperature, and used them to estimate heat strain, fatigue, or heat stress. The study presented in P14 is the paper that includes not only heat but also other aspects that might be affected by changes in climate conditions, such as dust and UV radiation (43).

The secondary data papers differ from the first category in terms of the year of publication. The six papers were published in the last 4 years, the oldest from 2021, showing that this approach is new when dealing with the climate issue on occupational health. Another difference is that this type of study uses data on occupational incidents that already occurred to investigate how climate affected it, while the objective of the first category of papers is to predict and prevent accidents and illnesses from happening. Therefore, the scenario changes for each category to develop a similar study to the one found in this review. The first requires access to a workplace and devices to gather information, while the second depends on records of occupational injuries or diseases.

Regarding the place of study, we screened 6 papers from United States of America (33–35, 37, 47, 52), 6 from Portugal (39, 42, 43, 46, 49, 50), 4 from South Korea (41, 42, 44, 48), 2 from China (32, 38), 1 from Hong Kong (51), 1 from Japan (36), 1 from India (45), 1 from Netherlands (31), 1 from United Kingdom (30), and 1 from Spain (53). We can notice that there is a trend of more developed countries, mainly from the world north, with four countries from Europe. Not one country from Latin America or Africa has been included in this review, which are countries vulnerable to climate change and increasing temperatures (54, 55).

## 5 Agenda for future research

**Figure 2:**
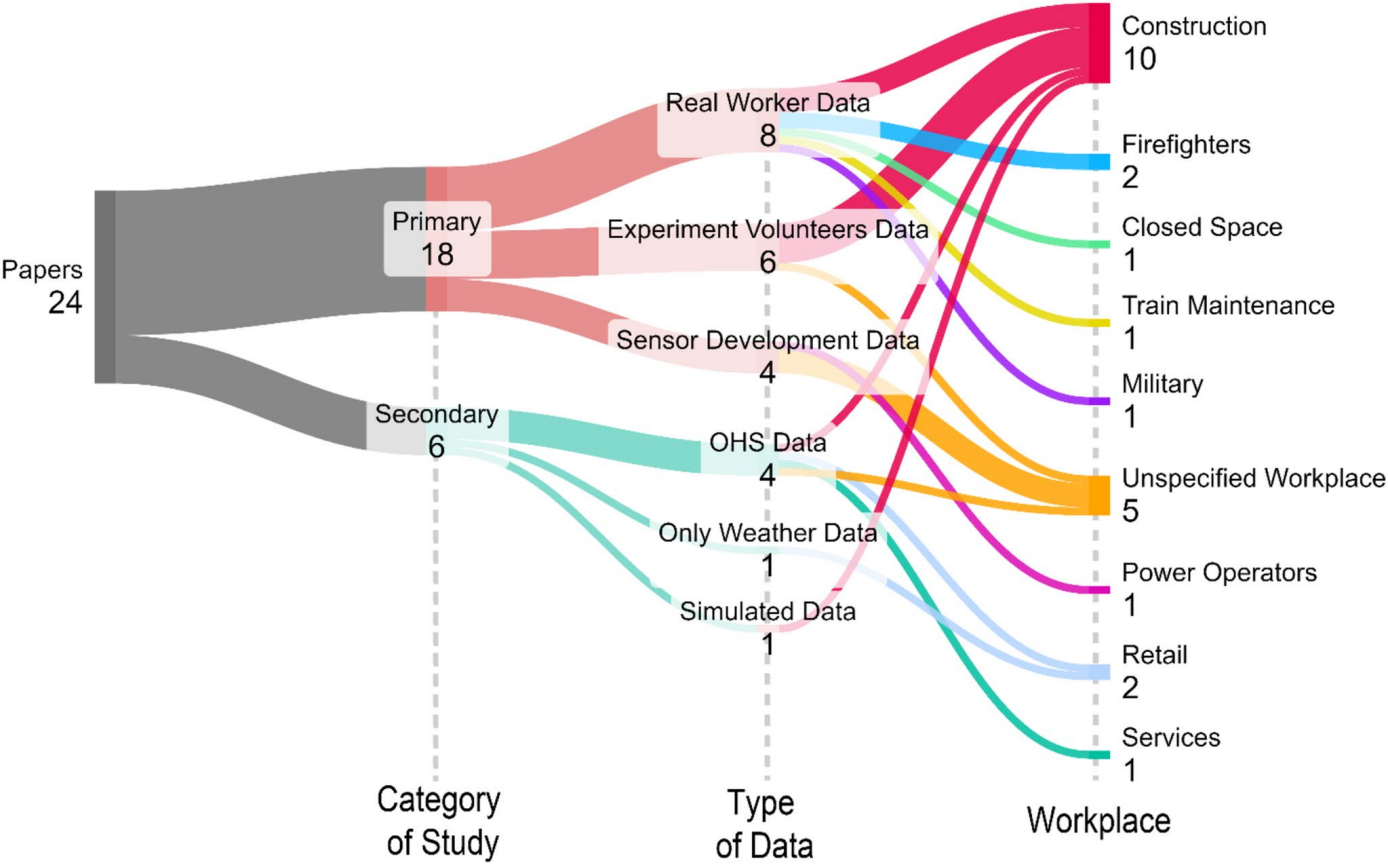
Classification of the findings according to category of study, type of data, and workplace studied.

Considering the findings of this systematic review, mainly that the research in this context is very recent, we built an agenda with some suggestions and possible paths to guide future research in the area. We developed a Sankey Chart, presented in Figure 2, to illustrate the previously discussed findings and guide the development of this research agenda. The ways to investigate the effects of climatic variables on OHS are divided into two possibilities regarding the type of data available to explore, according to the two categories: primary and secondary data.

Each path in this bifurcation has three types of data used; in primary data, there is either the use of real workers’ data or data from experiments with volunteers, or there is only the discussion regarding the development of sensor and wearable devices, with no real physiological data collection. On the other hand, when it comes to secondary data, most papers used some occupational health and safety data, such as injury records. In contrast, the others either used only meteorological data or simulated data to be used. We can also classify the studies based on the workplace focus of the research. These types of data collection for the two categories are examples of successful approaches and could guide future research. However, the workplace is quite the opposite; we believe that the more diverse workplaces are the focus of this type of research, the more we can comprehend the extent of climate strain on occupational force and health. This section will delve into these two main paths and discuss future research for them.

### 5.1 Research agenda for primary data studies

Primary data studies require access to occupational settings and real-time data from the environment and the workers. The use of sensors may be required to collect data, especially when it comes to gathering personal, physiological data to estimate the effects of heat stress on the well-being of workers. These characteristics may present some limitations. First, there needs to be a workplace open to this kind of research that allows collecting personal worker data. An alternative to this would be to collect data from volunteers, not precisely the workers, and develop an experiment to simulate the activity from that work setting, as done by P06, P11, P12, and P15. Another alternative would be to simulate data to test the sensor system, as done to test the system presented by P13, P14, and P17. Then the second limitation regards the tools needed to gather the data: sensors and wearable devices, which are not always available to every workplace or research team. Some authors developed their sensor systems, while others used more popular and accessible devices, such as smartwatches like the Apple Watch.

One characteristic of primary data is that the conclusions it achieves are restricted to the specific workplace and work settings being studied, which in most cases are confined to a single type of work at a time. For example, P01 focuses on military work (30), P02 on firefighters (31), P04 on construction workers (33), and P09 on closed spaces workers (38). Therefore, the results are limited to these specific scenarios. This characteristic represents gaps of research for every other work environment yet to be discussed, especially the ones most vulnerable to climatic variables, such as outdoor work or heavy-loaded activities, e.g., agriculture (6). To fill this gap, future research may need to adapt the sensors and wearable devices to fit into each occupation studied while also adapting what kind of data is relevant for each scenario, considering that each workplace differs from others.

Regarding data processing, there is a solid trend of using supervised machine learning to deal with this data, applying classification and regression algorithms, mainly SVM, RF, KNN, XGBoost, and MLP. This trend is similar to findings on the application of machine learning in occupational safety and health, where SVM was highlighted as one of the most used algorithms, along with Decision Trees and Naive Bayes (17). This shows that there is room to both follow the same path and improve the results found with these common techniques, but also there are other techniques that could be further explored, such as Decision Trees and Naive Bayes, which were used on 4 (30, 33, 36, 45) and 3 (36, 40, 43), respectively. Beyond these known supervised methods, some unsupervised methods could also be applied, such as clustering, which was only used by one paper (40). These represent possibilities to expand the literature. The comparison of other methods to these classification and regression algorithms commonly used could provide a more comprehensive view of how machine learning can be used and improve this type of research.

### 5.2 Research agenda for secondary data studies

Unlike primary data studies, secondary data studies do not need to visit workplaces and collect data. They use historical data, such as records of occupational accidents and injuries, such as the Korean national construction accidents database (48) or heat-related injuries (52), or meteorological information, such as Portugal national weather database (49, 50) or online platforms such as WorldClim (53). However, to achieve a successful result when dealing with secondary data, the research relies heavily on the availability of relevant and detailed data, the quality of the information, and the source’s reliability. This can be a limitation when open data is unavailable or not detailed enough to generate insights.

It is essential to highlight that this kind of data does not restrict the research to a single type of work; it can be generalized to all workplaces related to that occupation with records within the available database. For example, Lee et al. (48) tackled the problem in the national scenario of Korean construction, while (49) focused on the retail sector of Portugal. Also, studies using this information can generate results that consider data from the entire country or region, such as (52), that included every occupation recorded in the United States HRI database. Future research is not restricted to these occupations or locations. It should be expanded to other countries and regions, especially the most vulnerable to climate change in the global south, such as Latin America and Africa (6, 54, 55).

We identified a trend where the techniques used in this type of study were mainly related to the selection of features, with a trend of using methods such as FFS to select the most influential climatic variables on the occurrence of occupational accidents and injuries. These selection techniques may be combined alone or with supervised classification algorithms, such as SVM, RF, and Naive Bayes. Similar to what was mentioned for the primary data studies, these techniques could be used in more studies to improve the results and solidify these techniques as valid for this context; however, they do not prevent researchers from using and combining different machine learning methods.

## 6 Final considerations

This study aimed to investigate existing papers that explore the impacts of climatic variables on the occurrence of accidents and injuries and other occupational health and safety aspects and use some machine learning techniques to do so. This study combines three major subjects and investigates their combination: climate change, occupational health and safety, and machine learning. For each subject, we had papers from which we took inspiration and used to guide our research. For example, Rolnick et al. (25) showed us the opportunity to use machine learning to explore the impacts of climate change; however, it did not focus specifically on the impacts on occupational well-being. Pradhan e al. (10) did approach the impacts of climate change on workers but did not use any computational tool or method to study the data. Reis et al. (22) used ML to explore occupational accidents but did not consider anything related to climate. Therefore, with our paper, we aim to expand on what was done by these three papers while also filling this gap regarding the unification of the three subjects.

Our systematic review found 24 papers from international journals and conferences, most of them published in the last 5 years, with the oldest being from 2013. Therefore, this topic is new for scientific publications and has gained more popularity over the last couple of years, with 8 papers published in 2024. The present review was done in August 2024, meaning that more papers about the topic might be published until the end of the year. The results were categorized according to the data type used in the study, with primary and secondary data being the two options. Most of the papers used data actively collected by the authors; in all of them, it was done using wearable sensors, some of them using real workers in their experiments, while others used volunteers and students to simulate work activities. The data collected through sensors was processed through algorithms that used different data analytics approaches. This allowed an understanding of the workers’ physiological conditions and stress levels, culminating in improved real-time surveillance of occupational well-being.

The second category used data gathered from secondary sources, such as national occupational accidents and meteorological databases, and the data analytics approaches were used to understand this large volume of data, investigate existing relationships between the variables, or even identify the most relevant features that could be used to improve the development of heat-related risk predictive models. Although the data used in these studies were not gathered in real time, this approach is still relevant. It can help us better understand the behavior of occupational accidents and injury, and by adding the new layer of information of climatic variables, we can identify behaviors that were not yet perceived, and the use of machine learning techniques can be used for this exact purpose. Diverse work settings were studied, with construction workers being the most frequent in primary and secondary categories. Heat was the most explored factor that impacts occupational health, with one paper also tackling other issues that might be enhanced by climate change, such as dust and UV radiation.

Our findings point toward a significant trend in research where the most used approach is to collect physiological data through wearable sensors. We discussed the limitations and possibilities that accompany this trend in our research agenda, which includes the difficulty of finding an open workplace to apply this type of research, which requires access to personal data and might even involve invasive forms of data collection, with digestive pills to monitor core temperature, as seen in some screened paper. Also, it requires the gathering data device to work. It might need to be developed by the researchers, as seen in a few examples in this review, or even with existing devices, representing a financial cost to the research. In the research agenda, we also discussed some limitations regarding the secondary data, mainly affected by the availability and quality of occupational health and climate databases.

Future research regarding this topic could expand in three paths. The first uses the same methods and machine learning tasks but applies them to different work scenarios, considering the specificities of extra workplaces, such as agriculture or other outdoor occupations. The second path is to use different methods not yet used or little discussed, such as unsupervised machine learning or clustering tasks, to compare the results with the findings in the papers in this review. The third is regarding the expansion of this type of study to other locations, such as Latinos, Africans, third world, and the world south countries, that are mostly affected by climate change.
